# Response to: “Does ACPA-negative RA consist of subgroups related to sustained DMARD-free remission and serological markers at disease presentation” by Masi and Fleischmann

**DOI:** 10.1186/s13075-020-02152-9

**Published:** 2020-03-20

**Authors:** Debbie M. Boeters, Annette H. M. van der Helm–van Mil

**Affiliations:** 1 Department of Rheumatology C1-R, Leiden University Medical Center, PO Box 9600, Leiden, 2300 RC the Netherlands; 2 Department of Rheumatology, Erasmus University Medical Center, Rotterdam, the Netherlands

With interest, we read the letter of Masi and Fleischmann [1], and we are pleased with the interest in our study, in which we observed that subgroups can be identified within ACPA-negative RA when studying serum markers at disease presentation [2]. Masi and Fleischman had several comments.

First, they noted that the EAC started in 1993 and that not all RA patients in the cohort were studied, but only those of the most recent inclusion period (2011–2016). This is correct. Sustained DMARD-free remission was infrequent in the past and has become more frequent over time, with the highest frequency in the most recent inclusion period [3, 4]. As the outcome should be prevalent in order to have sufficient power to detect associations, we decided to determine serum markers in all RA patients that were consecutively included in this most recent inclusion period.

Second, Masi and Fleischmann mentioned that the results were flawed because age was neglected as potential confounder. The authors were incorrect. Potential confounders were evaluated. Subsequently age, and also swollen joint count and the presence of rheumatoid factor, were added as adjustment factors in a multivariable analysis. After these corrections, the association remained present and therefore age (or confounders linked to age) had not biased our results.

Finally, and perhaps most importantly, the authors noted that it was unexpected that we observed that higher MBDA scores at diagnosis associated with a lower risk to achieve sustained DMARD-free remission over time in ACPA-negative RA, because some previous studies reported that high MBDA scores related to higher disease activity scores in cross-sectional analyses and that low MBDA scores were predictive of achieving DAS-remission, as was recently shown by Fleischmann [5]. It seems that this misunderstanding is caused by confusing two different study aims. Fleischmann evaluated the MBDA score for the purpose for which it was developed, which is disease monitoring. We, in contrast, had a different and more basic aim. We wanted to increase the comprehension on the mechanisms underlying the development of sustained DMARD-free remission, and in line with this, to explore whether patient characteristics at diagnosis are related to the ability to achieve this long-term outcome. In previous studies, we observed that several markers of inflammation measured at diagnosis (swollen joint counts, C-reactive protein, and MRI-detected joint inflammation), poorly relate to this long-term outcome [4, 6]. As C-reactive protein is just one of many markers of inflammation present in the circulation, we now measured other inflammatory markers. The MBDA score is a tool that combines several markers and was used for practical reasons, but any other array that combines several proteins would have been equally interesting to start with. Our data is the first supporting that ACPA-negative RA consists of several subsets with differences in the long-term outcome that can be identified at the time of diagnosis. This finding is important and incentive to perform subsequent studies. Ideally, more inflammatory markers should be measured than the 12 markers that were studied now, in order to find the combination of markers with the highest discriminative ability. In addition, results need to be validated in other sets of ACPA-negative RA patients with long-term follow-up data. Hopefully, further studies on proteomics (possibly also including expression of other markers) will be helpful in differentiating ACPA-negative RA in clinically relevant subsets.

## Data Availability

Data are in published article.
