# Correction to: Response to: “Does ACPA-negative RA consist of subgroups related to sustained DMARD-free remission and serological markers at disease presentation” by Masi and Fleischmann

**DOI:** 10.1186/s13075-020-02201-3

**Published:** 2020-05-04

**Authors:** Debbie M. Boeters, Annette H. M. van der Helm–van Mil

**Affiliations:** 1 Department of Rheumatology C1-R, Leiden University Medical Center, PO Box 9600, Leiden, 2300 RC the Netherlands; 2 Department of Rheumatology, Erasmus University Medical Center, Rotterdam, the Netherlands

**Correction to: Arthritis Research & Therapy (2020) 22:55**

**https://doi.org/10.1186/s13075-020-02152-9**

Following publication of the original article [1], the authors reported an error.

In the last paragraph (first sentence) of the article it states that we have shown that “... higher MBDA scores at diagnosis associated with a lower risk to achieve DMARD-free remission over time...”, however it should be “… higher MBDA scores at diagnosis associated with a higher risk to achieve DMARD-free remission over time...”
